# Full Endoscopy Combined with Allogeneic Bone Grafting for Benign Spinal Lesions: Technical Notes and Preliminary Clinical Results

**DOI:** 10.3390/jcm12082990

**Published:** 2023-04-20

**Authors:** Cong-Gang Liao, Wen-Ge He, Qi-Chang Li, Qiang Ren, Jia-Nan Zhang, Liang-Jie He, Xiao-Juan Zhang, Liang Chen

**Affiliations:** 1 Department of Bone and Soft Tissue Oncology, Chongqing University Cancer Hospital, Chongqing 400030, China; 2 Chongqing Key Laboratory of Translational Research for Cancer Metastasis and Individualized Treatment, Chongqing University Cancer Hospital, Chongqing 400030, China

**Keywords:** percutaneous spinal endoscopy, benign spinal lesion, allograft bone graft, minimally invasive surgery

## Abstract

Benign lesions of the spine include benign tumors and tumor-like lesions of the spine, which usually occur in the thoracic and lumbar vertebrae. The incidence rate is low, accounting for about 1% of primary bone tumors. Few cases of endoscopic treatment of benign spinal lesions have been reported in the literature. Here, we introduce a new surgical technique using full endoscopy and allogeneic bone grafting to treat benign spinal lesions. All patients in this study successfully underwent the operation, and their pain was significantly relieved postoperatively. The patient VAS scores decreased from 3.07 ± 0.70 preoperatively to 0.33 ± 0.49 at the last follow-up visit (*p* < 0.05). The mean total blood loss (including drainage blood) was 16.67 ± 6.98 mL. The mean operative time was 63.33 ± 7.23 min. No patients developed numbness in the corresponding segmental distribution after surgery, none of the patients had serious postoperative complications, and none had focal recurrence during follow-up requiring reoperation. Patients reported symptom relief throughout the whole follow-up period. We believe that endoscopic surgery preserves the ligaments and soft tissues around the vertebral body, and that this technique is feasible with minimal trauma, rapid recovery, and good outcomes at short-term follow-up. This minimally invasive treatment modality offers a new option for the treatment of patients with benign spinal lesions.

## 1. Introduction

Benign spinal lesions include aneurysmal bone cysts, osteoid osteomas, and fibrous dysplasia, which tend to occur in the thoracic and lumbar spine. The incidence is low, accounting for approximately 1% of primary bone tumors [1,2]. They are usually found incidentally during physical examination, but local pain, nerve compression, and spinal deformity may also occur as the disease progresses [3]. Patients with benign spinal lesions that are asymptomatic and not suspected of malignant or aggressive behavior can be followed up periodically to observe the progression of the lesion. If the lesion is symptomatic, causing pain, compression of adjacent nerves, or even spinal deformity, surgical treatment is required [4].

The management of benign spinal lesions is still a challenge for clinicians due to a lack of evidence-based treatment strategies. Regular follow-up observations are indicated for asymptomatic patients with minor bone damage. However, in patients with symptomatic benign lesions, benign spinal lesions, although not metastatic, can lead to severe neurological deficits and often require surgical intervention [5]. The main surgical method for benign spinal lesions is the resection of lesion tissue and stability reconstruction. Because the spine is positioned far away from the body surface and the surrounding anatomical structures are complex, extensive soft tissue dissection is required, resulting in greater trauma, which can damage the normal physiology of the spine and lead to a series of complications.

Our team adopted “vertical anchoring technology” in the beginning of this surgery, which allows clinicians to safely and accurately reach the lesion site, providing a safe and effective minimally invasive surgical route [6,7]. In this method, we use a high-speed grinding drill under a full endoscope to grind the lesion down to the normal bone on the inner wall of the tumor cavity, use a radio frequency electrode to carry out internal thermal ablation of the lesion, and use concentrated iodophor and anhydrous ethanol to carry out chemical treatment of the tumor cavity, so as to minimize recurrence and damage to the normal spinal structure [8]. In the last step of the surgery, the cavity is filled with allogenic bone. In this study, we propose technical notes and short-term follow-up reports to illustrate the method of treating benign spinal diseases with full endoscopy combined with allogeneic bone transplantation.

## 2. Materials and Methods

### 2.1. Preoperative Preparations

The chief surgeon began percutaneous endoscopic spinal surgery in 2010, and all authors have extensive experience in percutaneous endoscopic spinal surgery, including the foraminal or interlaminar decompression approach for lumbar stenosis and discectomy for lumbar disc herniation. Before we began applying full endoscopy combined with allogeneic bone grafting for benign spinal lesions in 2018, we preliminarily performed the technique at different spinal levels in three cadavers.

### 2.2. Surgical Technique

#### 2.2.1. Surgical Instruments

The spinal endoscopic surgical instruments were made by Spinedos, Germany, and included an endoscope (7.5 mm outer diameter, 6.9 mm inner diameter), endoscopic sheath, 3 mm diameter high-speed grinding drill (variable direction grinding head), catheter inner ring saw, radiofrequency ablation electrode, 2 mm diameter Kirschner needle, gelatin sponge and homogeneous bone (Daqing Biological Co., Ltd., Chongqing, China).

#### 2.2.2. Surgical Procedure

**Figure 1:**
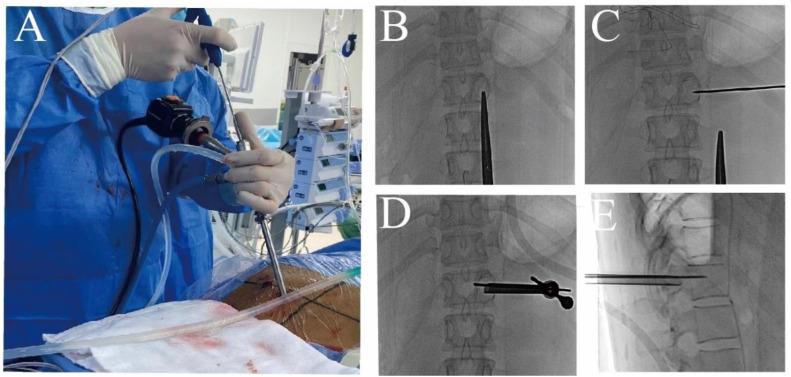
(**A**) The primary surgeon performing the endoscopic procedure. (**B**) Locating and marking of the diseased vertebral body. (**C**) Using a 2 mm Kirschner needle to puncture the vertebral body lesion. (**D**,**E**) Using the vertical anchoring technique to place endoscopic work channels and instruments, and perform the surgery.

**Figure 2:**
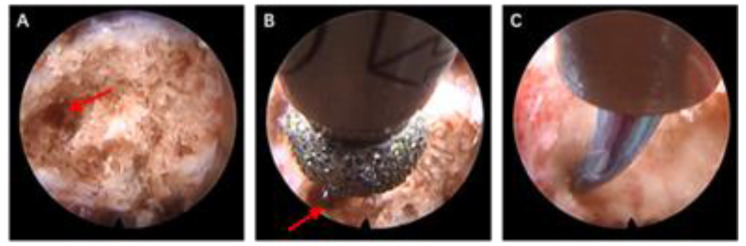

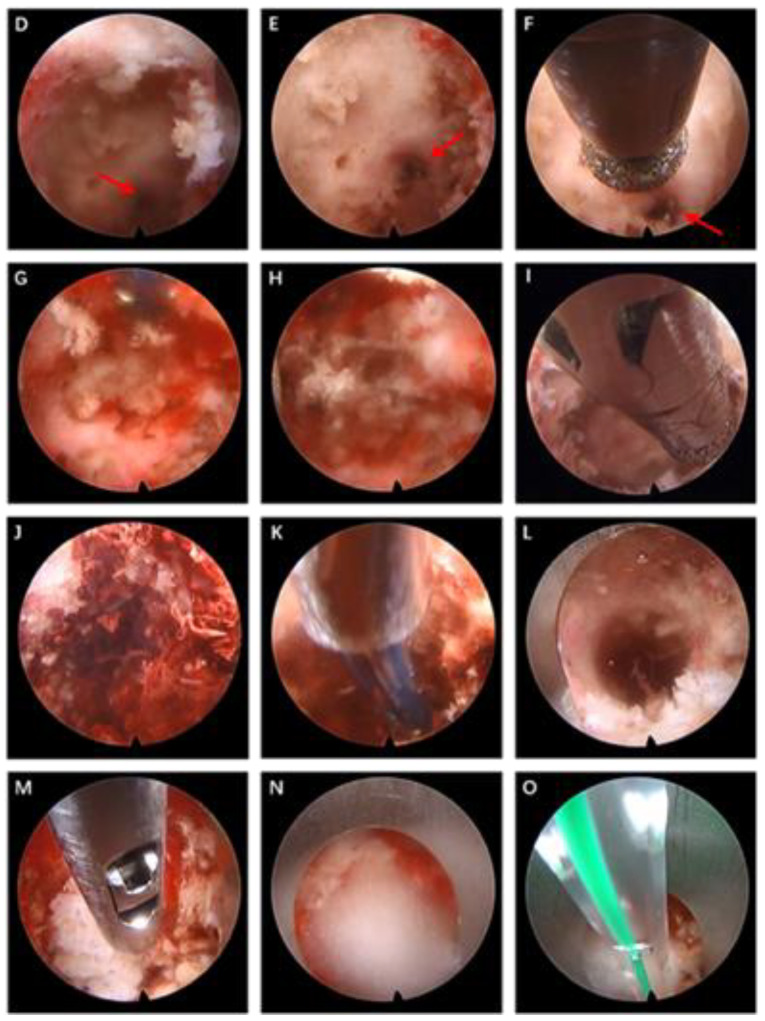
Operation procedure. (**A**,**B**) Abrasion along the vertebral arch toward the vertebral body using a 3 mm abrasive drill with the kerfing needle anchorage point (red arrow) as a marker; (**C**) radiofrequency electrode ablation of the intradiscal tumor wall; (**D**–**F**) the kerfing needle anchorage point (red arrow) remained visible during the abrasion along the vertebral arch toward the vertebral body; (**G**,**H**) rich osteopontic blood supply in the vertebral body; (**I**) the direction of the abrasive head was adjusted to deal with the intradiscal lesion; (**J**) concentrated iodophor and anhydrous ethanol were used after chemical inactivation of the internal vertebral body; (**K**) radiofrequency ablation of the intravertebral tumor wall; (**L**) the opening on the vertebral arch root plate, approximately 3–4 mm in diameter; (**M**) filling with homogeneous allograft bone; (**N**) the opening on the vertebral arch root plate was sealed using a gelatin sponge; (**O**) a drainage tube was left in place along the working channel.

For benign lesions of the vertebral body, general anesthesia was used for the operation. After satisfactory anesthesia, the patient was placed in a prone position on the operating table and an arch was placed to avoid abdominal pressure. DSA fluoroscopy was used to locate the lesion side of vertebral body, and a mark was made at approximately 2–3 cm above the lateral projection of the vertebral arch. A transverse incision of approximately 0.8 cm was made at the mark, and a 2 mm kerfing needle was used to puncture the lateral arch of the lesion and anchor the kerfing needle along the arch to the interior of the vertebral lesion using a bone hammer. A step-by-step dilating catheter was used to dilate the working channel along the Kirschner needle puncture path to the bony surface of the vertebral plate, and an endocannabinoid saw was placed (Figure 1). After fluoroscopic confirmation of a satisfactory position, an annular mark was made on the bony surface of the vertebral plate. The annular saw was removed, the kerfing needle was removed, and the endoscope was placed with the “target site” visible under the scope. After clearing the soft tissues around the vertebral plate, the central part of the “target point”, the anchoring point of the Kirschner needle, was centered, and a 3 mm diameter high-speed grinding drill was used to grind away the lesion from the pedicle toward the vertebral body. Since the tip of the needle was anchored at the posterior edge of the vertebral body, a microscopic marker of the anchoring point of the needle was always visible during the grinding process along the arch to the posterior edge of the vertebral body, ensuring safe and accurate progression of the grinding drill to the interior of the vertebral body through the arch. After the lesion was cleared from the vertebral body and arch using nucleus pulposus forceps, the tissue was frozen and sectioned to confirm that it was not a malignant tumor. The lining of the vertebral body and pedicle cavity was removed using a grinding drill until normal bone was seen microscopically, and the cavity was chemically inactivated using concentrated iodine and anhydrous ethanol after radiofrequency electrode ablation of the cavity lining for 5–10 min. After careful inspection of the vertebral body for residual focal tissue, the cavity was filled with homogeneous bone along the working channel and the opening was sealed with a gelatin sponge. One drainage tube was left in place along the working channel, the skin was closed with one full suture and the drainage tube was fixed, and the procedure was completed (Figure 2).

#### 2.2.3. Postoperative Management and Follow-Up

Postoperative analgesic medications were routinely administered and discontinued within 24 h postoperatively to assess patient pain improvement. Absolute bed rest was maintained for 24 h after surgery, and drainage tubes were removed. On the second postoperative day, the head of the bed was gradually raised with a rigid brace, and, if there was no significant discomfort, the patient was allowed to move to the ground appropriately.

### 2.3. Analysis of Clinical Results

From January 2020 to May 2022, a total of 15 patients with benign spinal lesions were enrolled, and all patients were treated with “full endoscopy combined with allogeneic bone grafting”. All patients were followed up for more than 12 months. All enrolled patients signed relevant surgical consent and informed consent forms. General information, diagnosis, estimated blood loss, operative time, and complications were evaluated. The visual analog scale (VAS) was used for preoperative and postoperative evaluation. This study was approved by the Ethics Committee of the Affiliated Cancer Hospital of Chongqing University, with informed consent from the patients. Statistical analysis was carried out using SPSS 22.0 online at https://spssau.com/indexs.html?372257& (accessed on 20 December 2022). Quantitative data are expressed as average ± standard deviation. Student’s *t* test was used to compare the differences between the two groups. *p* < 0.05 was considered statistically significant.

## 3. Results

A total of 15 patients (6 males and 9 females; mean age 21.8 ± 4.3 years) were recruited from January 2020 and were included in this study. The mean follow-up time was 18.0 ± 5.1 months. All 15 patients had benign spinal lesions that were treated with full endoscopy combined with allogeneic bone grafting. Three patients had aneurysmal bone cysts, eight patients had osteoid osteoma, and four patients had fibrous dysplasia (Table 1).

VAS scores improved significantly after surgery. The VAS scores decreased from 3.07 ± 0.70 preoperatively to 0.33 ± 0.49 at the last follow-up visit (*p* < 0.05). No patients had disease recurrence during postoperative follow-up. The mean total blood loss (including drainage blood) was 16.67 ± 6.98 mL. The mean operative time was 63.33 ± 7.23 min (Table 2).

No patients developed numbness in the corresponding segmental distribution after surgery, none of the patients had serious postoperative complications, and none had focal recurrence requiring reoperation during follow-up. At the last follow-up, all 15 patients were satisfied with the bone graft filling. 

**Figure 3:**
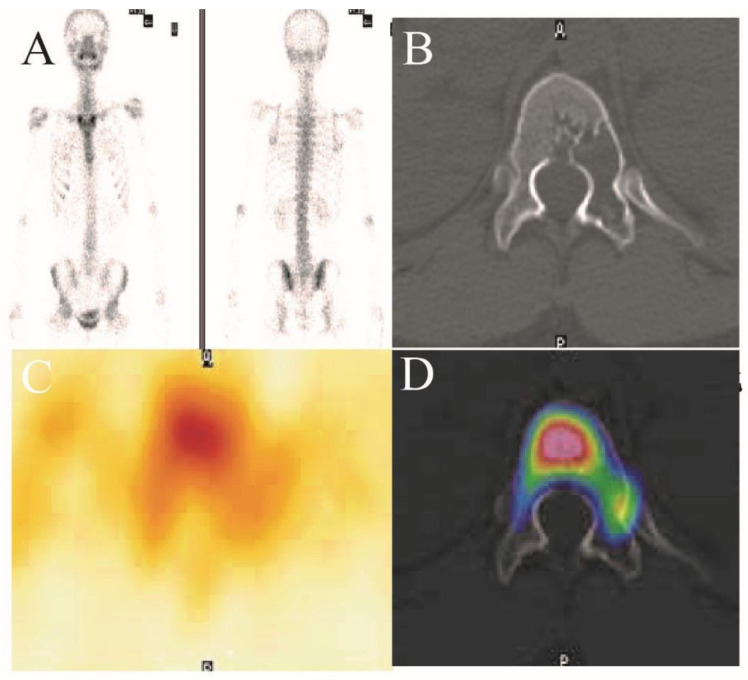
(**A**–**D**). Whole-body bone imaging, CT image, SPECT image, and SPECT/CT fusion image suggesting that the left posterior portion of the T12 vertebral body and the left accessory bone were destroyed, with clear borders and sclerotic margins, without visualizer concentration.

**Figure 4:**
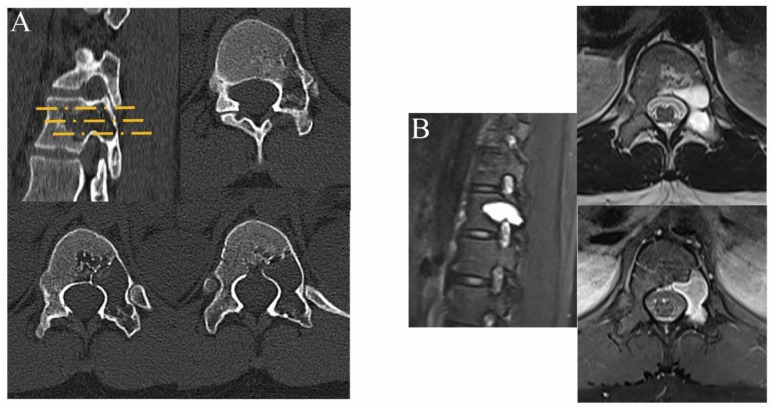
(**A**) CT scanning showed that part of the vertebral body and pedicle on the left side of the T12 vertebral body showed slight expansive bone destruction, the marginal bone was slightly sclerotic, the boundary was clear, and there was no periosteal reaction. (**B**) MRI images showed cystic high signal shadows in the left part of the T12 vertebral body and in the pedicle of the vertebral arch, which could be evenly enhanced so no soft tissue mass shadow was seen and the paravertebral space was clear.

In all of these cases, we present a typical case of a patient with a T12 vertebral benign lesion who was treated with a percutaneous endoscopy combined with allogeneic bone grafting in 2021. The patient, a 17-year-old female, was admitted to the hospital with “back pain for 3 years and T12 vertebral lesion for 1 year”. The patient had persistent nonmechanical pain in the lower back as the main symptom (VAS score 4), without spinal cord or nerve involvement, with a long duration of disease and poor relief with conservative treatment. Preoperative imaging suggested a benign osteolytic lesion in the T12 vertebral body, located within the C level of Weinstein-Boriani-Biagini (WBB) classification 4–5 (Figure 3 and Figure 4) [9]. A preoperative spinal instability neoplastic score (SINS) score of 7 (3 for lesion located at the junction, 1 for nonmechanical pain, 2 for osteolytic lesion, 0 for spinal sequence alignment, 0 for no vertebral body collapse and <50% vertebral involvement, and 1 for accessory involvement on one side) was determined in the assessment of acceptable spinal instability [10]. Full spinal endoscopic scraping of the T12 vertebral body lesion and allograft bone grafting were performed under general anesthesia in July 2021.

**Figure 5:**
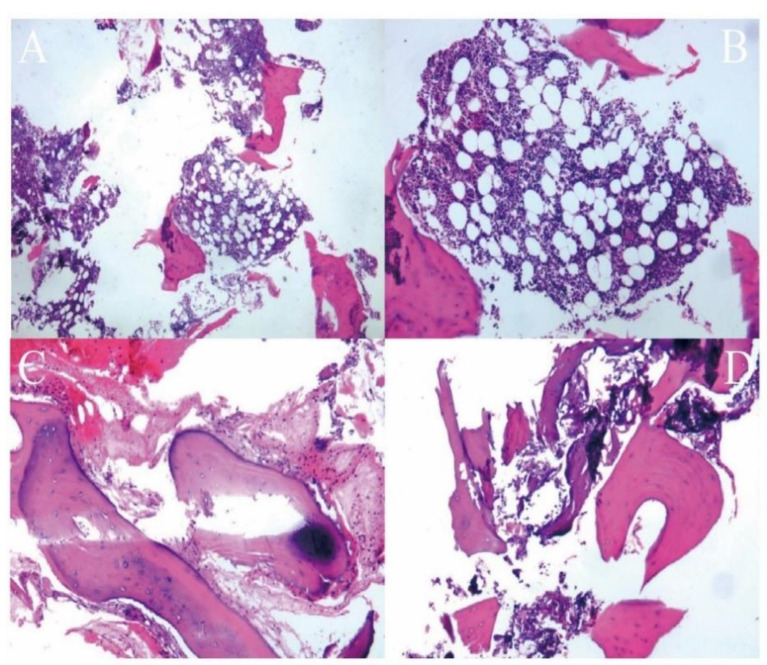
(**A**–**D**) Pathological findings suggested that no clear tumor cells were found, a little fibrous tissue proliferation was seen around the bone trabeculae, focal with minor inflammatory cell infiltration, and collagenization and ossification were seen in some areas. No obvious tumor tissue containing multinucleated giant cells (ABC features) was found.

**Figure 6:**
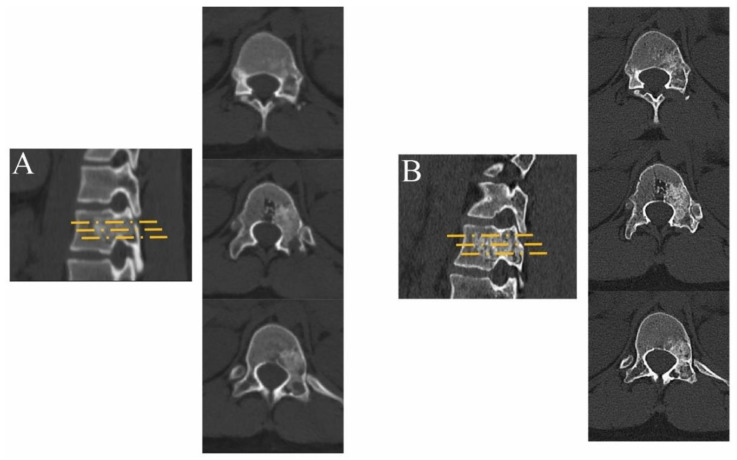
(**A**) Two days after the operation, CT showed that the lesion was completely cleared and the bone graft was fully filled. (**B**) Half a year after the operation, CT showed that the lesion area was completely filled with allograft bone, indicating that the bone graft was successful.

No tumor cells were found in the postoperative pathology results, and a diagnosis of aneurysmal bone cyst (ABC) was made by combining the imaging examination and clinical manifestations (Figure 5). The review imaging suggested complete scraping of the lesion and good placement of the bone graft. Six months postoperatively, imaging suggested good healing of the bone graft without recurrence (Figure 6). In this study, preoperative and postoperative images of some patients with benign spinal lesions, as shown in Supplementary Figure S1, showed successful bone grafting without recurrence.

At the 18-months postoperative follow-up, there was complete relief of back pain, a SINS score of 0, and good spine stability (Supplementary Figures S1–S3).

## 4. Discussion

Although aneurysmal bone cysts, osteoid osteomas, benign fibrous histiocytomas, and fibrous dysplasia are considered benign lesions, they are potentially aggressive and can lead to destruction of the bone and surrounding soft tissues [11]. Patients with benign spinal lesions that are asymptomatic and not suspected of malignant or aggressive behavior can be followed up periodically to observe lesion progression. If the lesion is symptomatic, causing pain, compressing adjacent nerves, or is even a spinal deformity, then surgical treatment is usually required. In the case of aneurysmal bone cysts within the vertebral body, due to the presence of important nerves, blood vessels, and other structures in the surrounding area, surgery is most often performed by removing the vertebral body in pieces or in its entirety. This involves extensive soft tissue debridement and stability reconstruction, which is traumatic and disrupts the normal physiological structure of the spine, so the development of a minimally invasive surgical approach represents a breakthrough in solving these problems.

With the development of spinal endoscopic techniques in recent years, the indications have expanded from degenerative spinal diseases to spinal infections and even spinal neoplastic diseases [12,13,14]. Depending on the number and size of working channels, spinal endoscopy can be divided into several different systems, including microendoscopic and percutaneous endoscopic surgery. Mehmet Zileli et al. used microendoscopic surgery for the treatment of osteoid osteoma of the C2 vertebral body, performing intraoperative lesion removal followed by fixation of the vertebral body using an internal fixation device. Three years of postoperative follow-up showed good cervical spine motion and complete recovery, which validated the feasibility of endoscopic surgical treatment in these cases [15]. In 2018, Shibuya et al. first reported a case of symptomatic benign spinal lesions of the lumbar spine treated using full spinal endoscopy [16]. The patient, who had predominantly localized pain symptoms and no spinal cord or neurological impairment, underwent full endoscopic debridement of the lesion and implantation of hydroxymethylapatite. In 2020, Xie et al. reported 11 cases of symptomatic benign spinal lesions of the lumbar and sacral spine treated with full spinal endoscopy, and all patients showed symptomatic relief with no recurrence during 1–3 years of follow-up [17]. In 2022, Kotheeranurak et al. reported treatment of a case of osteoid osteoma of the L3 vertebral body with no recurrence at 2 years follow-up [18].

Our research team has extensive experience in endoscopic procedures using full spinal endoscopy for degenerative spinal diseases [19,20,21,22,23]. We were aware of the various advantages of endoscopy, such as visualization of the operation, minimal surgical trauma, and rapid recovery, so we began to apply our endoscopic technique to benign spinal lesions. The treatment of benign spinal lesions focuses on removing focal tissue and reducing residual lesions, thereby reducing the probability of recurrence. Therefore, accurate puncture localization and surgical manipulation are important to reduce the harm caused to the patient by the procedure. Our team adopted the “vertical anchoring technique” for full spinal endoscopic surgery, which provides a safe and effective minimally invasive surgical approach by safely and accurately reaching the lesion [6,7]. The “anchoring technique” is a puncture technique that aids in the establishment of a working channel, which was first applied to the cervical spine and reported by our team in 2018. During endoscopic access establishment, a 2 mm Kirschner needle is used to puncture and anchor the bone surface at the target location, and a circular saw is placed along the Kirschner needle puncture path to mark the bone surface. The “target point” (consisting of the central “point” anchored by the Kirschner pin and the “ring” marked by the ring saw) is visible under the endoscope. This technique has been routinely used in full endoscopic surgery for degenerative diseases of the cervical and lumbar spine, and has two main advantages. One is that the kerfing needle is anchored on the bone surface during the establishment of the working channel, which can fix the working channel and avoid channel slippage, so as to reduce the number of fluoroscopic views required during the establishment of the working channel. Secondly, marking the “target point” under the endoscope can help the operator to quickly identify the target area and enhance the safety of the surgery. In the treatment of degenerative cervical spine diseases, endoscopic surgery combined with this anchoring technique shortens the operative time and reduces the number of intraoperative fluoroscopies compared to conventional endoscopic surgery, and has comparable efficacy to conventional endoscopic surgery.

The goals of treatment for benign spinal lesions are to improve symptoms, obtain a histological diagnosis, and prevent recurrence of the disease. Gibbs et al. concluded that complete removal of the surface of the tumor cavity to normal bone is necessary for recurrence prevention [24]. Xie et al. used full spinal endoscopy combined with radiofrequency to manage lesions, and all patients in their study showed symptomatic relief and no recurrence during follow-up [17]. High temperatures reduce tumor recurrence; therefore, in all our cases, after grinding the lining of the tumor cavity to normal bone, radiofrequency electrodes were used to ablate the cavity of tumor for 5–10 min. Ulici et al. reported that the treatment of aneurysmal bone cysts with anhydrous alcohol injection reduced the tumor lesions, suggesting that the foreign body inflammatory response was induced by anhydrous alcohol and that this indirectly stopped the process of ABC through vascular thrombosis and tissue ischemia [25]. Intraoperative iodine volts were also used to flush the focal tissue within the vertebral body, as free iodine in iodine volts can prevent surgical infection [26]. The above steps minimized the possibility of recurrence and surgical complications.

This was a retrospective study. The main symptom of all patients was back pain, so we considered that use of VAS scores was a simple and effective means to evaluate the effect of surgery. The VAS scores decreased from 3.07 ± 0.70 preoperatively to 0.33 ± 0.49 at the last follow-up visit (*p* < 0.05). According to this study, endoscopic surgery has less bleeding (average total blood loss was 16.67 ± 6.98 mL) and shorter operation time (average operation time was 63.33 ± 7.23 min). No patients developed numbness in the corresponding segmental distribution after surgery, none of the patients had serious postoperative complications, and none had focal recurrence during follow-up requiring reoperation. All 15 patients had no recurrence during the follow-up, and the pain symptoms were relieved satisfactorily.

Although fillers were not used after endoscopic treatment of the lesion in the study of Xie et al. [17], Shibuya et al. used hydroxyapatite for filling [16]. In our procedure, we filled with an allogeneic bone graft after removing the diseased tissue from the vertebral body. We believe that intravertebral bone grafting is necessary to increase the strength and stability of the vertebral body and reduce the risk of postoperative pathological fractures. Related studies have concluded that homogeneous allogeneic bone has the exact same composition, structure, and biomechanical properties as the patient’s own bone, which cannot be replicated by artificial bone [27]. In this study, all 15 patients who were followed up showed satisfactory bone grafts, which also showed that allogeneic bone was suitable for this technique.

Although our technology has achieved positive short-term results in the treatment of benign lesions of the spine, it still has some limitations. Not all patients are suitable for this technology, and it is applicable to small spinal lesions and mild nerve compression. However, open surgery is still needed for large tumors and severe nerve compression. Endoscopic surgery requires experienced doctors, and there may be problems associated with radiation exposure. A prospective, multicenter, and long-term study is needed to further explore the safety and effectiveness of the protocol.

## 5. Conclusions

In this study, our team adopted “vertical anchoring technology”, which enables the endoscope to reach the lesion safely and accurately. The use of a high-speed grinding drill, radio frequency electrode and anhydrous ethanol can minimize the recurrence and damage to the normal spinal structure. It should be emphasized that filling the cavity with allogeneic bone improves the stability of the vertebral body. It showed that full endoscopy combined with allogeneic bone transplantation is feasible for the treatment of benign spinal diseases. This technique has the advantages of short operation time, less bleeding and good operation effect. This minimally invasive treatment modality offers a new option for minimally invasive treatment of patients with benign spinal lesions.

## Figures and Tables

**Table 1:** Patient characteristics.

Characteristic	Value
Mean age (years)	21.8 ± 4.3
Sex	
M	6
F	9
Mean follow-up period (months)	18.0 ± 5.1
Diagnosis	
aneurysmal bone cyst	3
osteoid osteoma	8
fibrous dysplasia	4
Mean estimated blood loss (mL)	16.7 ± 6.9
Mean operative time (min)	63.3 ± 7.2
Postoperative complications	
Numbness	0

**Table 2:** Clinical characteristics data of all patients with benign spinal lesions.

Diagnosis	Location/WBB Classification	Age/ Gender	Conservative Treatment Time	Visual Analog Scale	Operative Time	Estimated Blood Loss	Follow-Up Period	Recurrence	Visual Analog Scale Score at Last Follow-Up
ABC	T12/sectors4-5, layer C	17/F	3M	4	55	10	18M	N	1
ABC	T10/sectors4-6, layer C	28/F	4M	3	70	15	24M	N	0
ABC	T1/sectors7-8, layer C	20/M	2M	4	50	15	18M	N	1
OO	T11/sectors4-5, layer C	22/F	2M	3	70	10	18M	N	0
OO	T12/sectors7-9, layer C	18/M	3M	2	65	10	18M	N	0
OO	T8/sectors7-8, layer C	21/F	5M	3	55	10	12M	N	1
OO	L2/sectors4-5, layer C	25/F	8M	4	60	20	18M	N	0
OO	L2/sectors4-5, layer C	30/F	2M	3	65	20	12M	N	1
OO	L3/sectors4-6, layer C	19/M	12M	3	75	20	24M	N	0
OO	L4/sectors5-6, layer C	19/F	12M	2	65	30	12M	N	0
OO	L4/sectors4-5, layer C	22/M	14M	2	70	30	24M	N	0
FD	T8/sectors4-5, layer C	23/M	10M	3	55	10	18M	N	0
FD	T11/sectors4-5, layer C	19/F	5M	3	60	10	12M	N	0
FD	L3/sectors4-5, layer C	16/F	3M	4	65	20	18M	N	0
FD	L5/sectors8-9, layer C	28/M	2M	3	70	20	24M	N	1

ABC, aneurysmal bone cyst; OO, osteoid osteoma; FD, fibrous dysplasia; WBB, Weinstein-Boriani-Biagini.

## Data Availability

The raw data acquisition will be made available by the authors, without undue reservation.

## Supplementary Materials

The following supporting information can be downloaded at: https://www.mdpi.com/article/10.3390/jcm12082990/s1.
