# Supplementary material for: Full Endoscopy Combined with Allogeneic Bone Grafting for Benign Spinal Lesions: Technical Notes and Preliminary Clinical Results
Source: J Clin Med. 2023 Apr 20;12(8):2990. doi: 10.3390/jcm12082990 (PMC10144314; doi:10.3390/jcm12082990)
Supplement: Supplementary file 1 [file jcm-12-02990-s001.zip › jcm-2234562-supplementary.pdf]

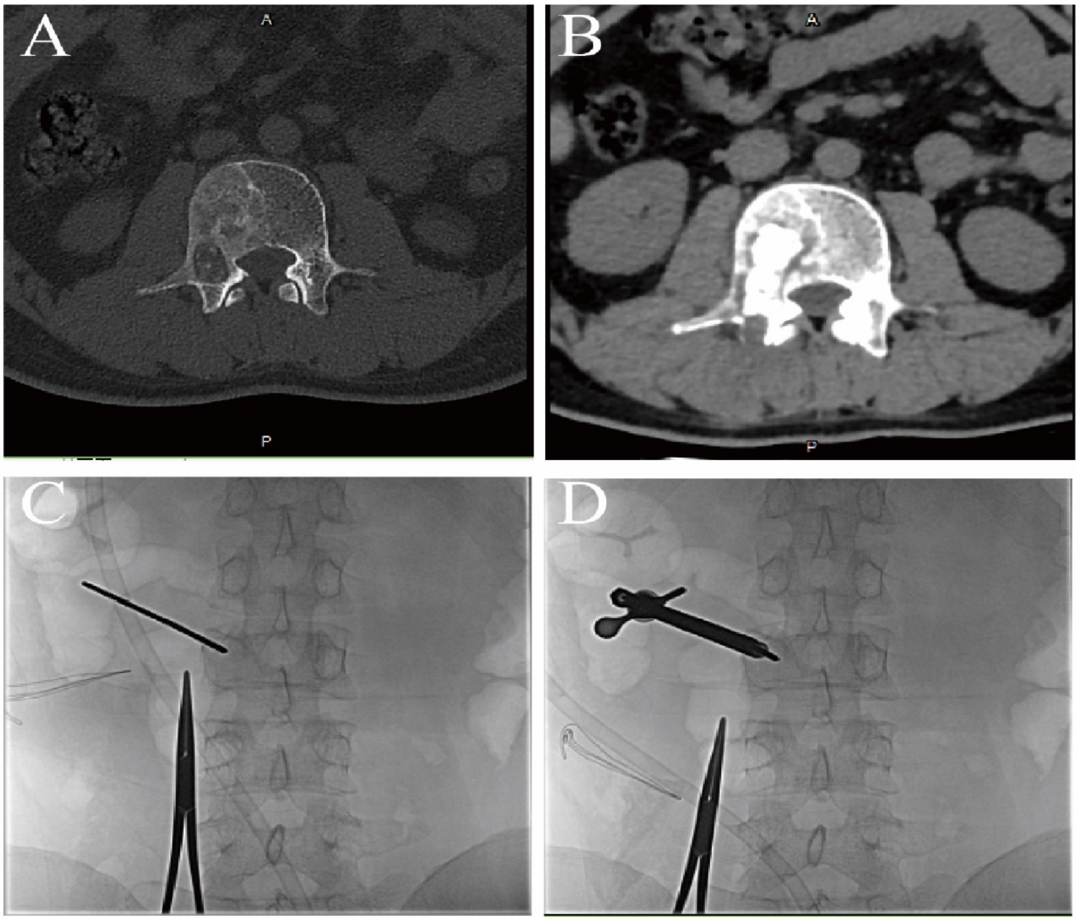

Supplementary Figure S1. (A–D) A patient with fibrous dysplasia of the L3 vertebra: preoperative and postoperative CT images, and intraoperative fluoroscopic puncture localization images.

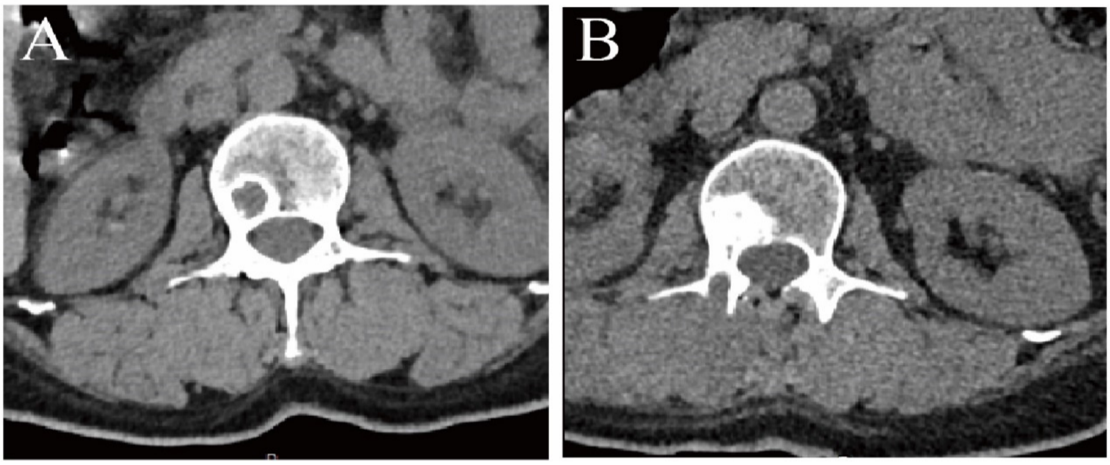

Supplementary Figure S2. In a patient with benign fibrous histiocytoma of bone in the L2 vertebral (A) preoperative CT images show a well-delineated intramedullary tumor pressing, dilating, thinning, and hardening into the cortical bone. (B) Postoperative CT images showed successful bone grafting after complete tumor curettage.

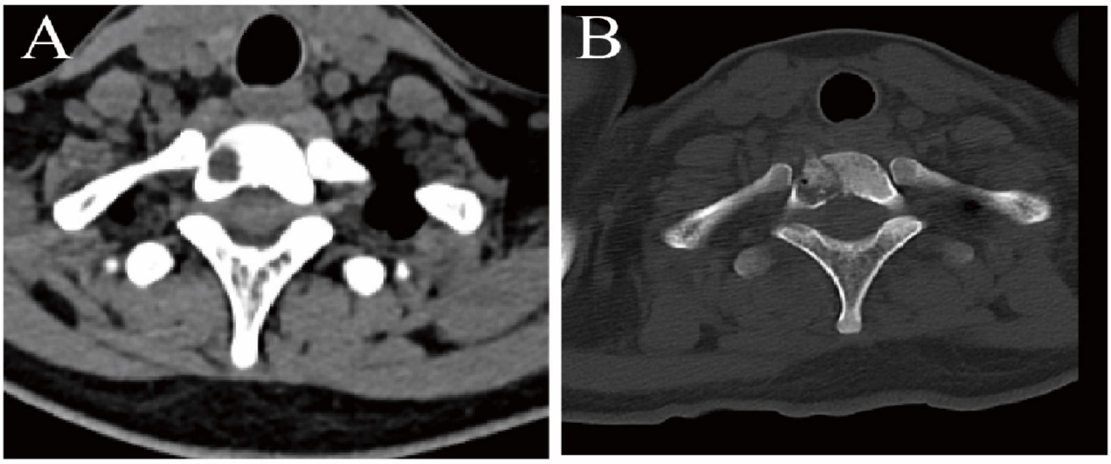

Supplementary Figure S3: In a patient with aneurysmal bone cyst in the T1 vertebral body. (A) Preoperative CT examination indicated intravertebral lesions. (B). Postoperative CT examination indicated intracranial shrinkage of vertebral lesion.
